# Symphony: A Framework for Accurate and Holistic WSN Simulation

**DOI:** 10.3390/s150304677

**Published:** 2015-02-25

**Authors:** Laurynas Riliskis, Evgeny Osipov

**Affiliations:** 1 Computer Science Department, Stanford University, 353 Serra Mall, Stanford, CA 94305, USA; 2 Department of Computer Science, Electrical and Space Engineering, Luleå University of Techno Luleå 971-87, Sweden; E-Mail: evgeny.osipov@ltu.se

**Keywords:** wireless sensor networks, emulation, sensors, simulations

## Abstract

Research on wireless sensor networks has progressed rapidly over the last decade, and these technologies have been widely adopted for both industrial and domestic uses. Several operating systems have been developed, along with a multitude of network protocols for all layers of the communication stack. Industrial Wireless Sensor Network (WSN) systems must satisfy strict criteria and are typically more complex and larger in scale than domestic systems. Together with the non-deterministic behavior of network hardware in real settings, this greatly complicates the debugging and testing of WSN functionality. To facilitate the testing, validation, and debugging of large-scale WSN systems, we have developed a simulation framework that accurately reproduces the processes that occur inside real equipment, including both hardware- and software-induced delays. The core of the framework consists of a virtualized operating system and an emulated hardware platform that is integrated with the general purpose network simulator ns-3. Our framework enables the user to adjust the real code base as would be done in real deployments and also to test the boundary effects of different hardware components on the performance of distributed applications and protocols. Additionally we have developed a clock emulator with several different skew models and a component that handles sensory data feeds. The new framework should substantially shorten WSN application development cycles.

## 1. Introduction

Simulations are the most widely used tools for analyzing the performance of protocols for communications networks. They are also used extensively to study the performance of wireless sensor networks (WSNs). However, WSNs have an important peculiarity that complicates simulation-based studies. Most (if not all) network simulators execute experiment scenarios in high-end machines but in WSNs, the limited resources of the network hardware are often a major performance-limiting factor. WSN simulations that do not account for hardware delays, time skew, delays associated with sensory data flows, and the execution model of the hardware's operating system therefore often produce unrealistic performance figures.

**Figure 1.**
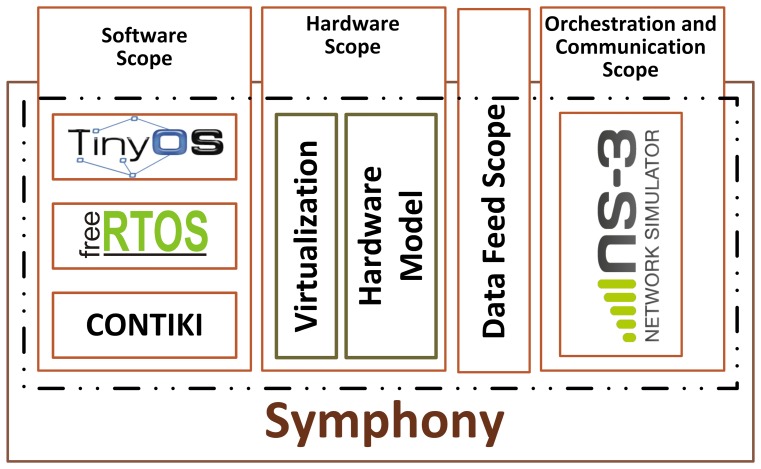
A high level architectural overview of Symphony.

This article describes a simulation framework known as Symphony (Symphony is released as open source and is available for download at https://bitbucket.org/Northshoot/symphony) that was designed for the testing and validation of WSN applications. The framework accurately reproduces the processes that occur inside real WSN equipment, including both hardware- and software-induced delays, and the dynamic flow of sensory data. Figure 1 shows its high level architecture. The overall purpose of Symphony is to provide a holistic framework in which WSN software can be developed and its functionality simulated in a single integrated development environment. In brief, when using Symphony, a WSN developer always has access to a ‘real’ implementation of their application in an OS that is used in WSN hardware such as TinyOS, FreeRTOS or Contiki. Symphony uses virtualization and hardware modeling techniques that allow the developer to work on a ‘real’ node while also smoothly integrating the real implementation of the application with a general purpose network simulator that enables extensive testing of its distributed functionality in a controlled and repeatable manner.

Technically, Symphony consists of four operating and programming scopes: a software scope, a hardware scope, a data feed scope, and an orchestration and communication scope.

The software scope provides the tools required to create a virtual image of an existing WSN operating system and a set of rules for doing so. The hardware scope consists of a set of models that accurately emulate the delays and energy consumption of various WSN hardware components. The data feed scope provides tools and models for making sensory data available to the virtualized node. Finally, the orchestration and communication scope is provided by the popular network simulator ns-3 (http://www.nsnam.org/), which enables the straightforward creation and execution of various simulated scenarios. Symphony thus bridges the gap between simulated and real WSNs. Its key features are that it:

1. Enables the user to experiment with the code base that would be used in a real deployment;
2. Preserves the execution model of the underlying operating system;
3. Accounts for the effect of hardware-induced delays on the performance of distributed applications and protocols;
4. Enables experimentation with a range of clock skew models;
5. Enables experimentation with several different applications and different WSN operating systems within a single simulation;
6. Provides a customizable level of simulation detail, ranging from fine-grained firmware emulation to system-level experiments;
7. Allows the user to investigate performance-related phenomena across the entire sensory data path.

The article is organized as follows. Section 2 provides a brief review of relevant previous work. Section 3 outlines the architecture of Symphony. Section 4 provides more details on the software scope. The hardware scope is detailed in Section 5. The data feed scope is presented in Section 6. The performance of Symphony is discussed in Section 7, and Section 8 summarizes the findings and concludes the article.

## 2. Previous Work

Simulations are the preferred tool for experimentation with communication networks for technical, logistical, and cost reasons. Following the emergence of WSN technology, various general purpose network simulators (e.g., ns-2 [1], ns-3 [2], Omnet [3], and Qualnet [4]) have been extended by the addition of WSN-specific frameworks. However, WSN technologies have two features that make their simulation more challenging than that of typical high-end communication systems: delays introduced by the use of low-end hardware components, and the peculiarities of the execution models used in the operating systems of those components. Extensive surveys of existing simulation tools have been presented by various authors (see [5–7] and references therein). In order to avoid unnecessary repetition, this article discusses only the most widely used existing tools and focuses on the problem of closing the gap between simulated and real WSN software as well as the unique features of Symphony that are listed in Section 1.

Over the last decade, numerous protocols for use in WSNs have been proposed in the literature. In most cases, their functionality was implemented and tested in artificial environments created inside general purpose network simulators. Details of these implementations are not generally available [8]. This situation has been criticized extensively [9–11]. As a result, many practitioners have been forced to implement protocols from scratch, highlighting the gap between simulator-specific implementations and implementation on real hardware platforms [12–15]. The remainder of this discussion deals exclusively with simulation tools that are supplied with mainstream operating systems.

Operating systems for WSNs generally follow one of three design paradigms: they may be event-driven (e.g., TinyOS [16]), threaded (e.g., Contiki [17]), or some mixture of event-driven and threaded (for detailed overviews of WSN operating systems, see [18–20]). While each paradigm has its pros and cons, operating systems of all three types are available on the market and the performance of a given set of distributed algorithms and communication protocols can vary dramatically depending on the choice of underlying OS and the composition of its software modules [21]. In this section, we focus on benchmarking Symphony's functionality against the simulation facilities supplied with the three most popular WSN operating systems: Contiki, TinyOS and FreeRTOS. Other operating systems are not considered either because their development has been abandoned or due to their proprietary code bases. The simulation tools provided with the currently used operating systems are primarily intended for simple debugging purposes. Previous attempts to increase their sophistication rapidly became outdated with the appearance of new versions of the relevant operating systems. Examples of such abandoned simulators that had similar functionality to Symphony in some respects include EmStar [22], which provided node virtualization and was discontinued in 2005; Atemu [23], which made it possible to perform simulations using real code (TinyOS) and was discontinued in 2004; Avrora [24], which provided precise timing models and was discontinued in 2009; and PowerTOSSIM [25], which enabled energy modeling and was discontinued in 2010. It should be noted that none of these extensions provided all of the features of Symphony or combined them in an integrated way.

Table 1 compares the functionality provided by existing WSN simulators to that of Symphony. When reviewing this table, one point relating to simulation tools that provide instruction-level emulation of software should be noted. Cooja is typical of such simulation environments in that it has an integrated microcontroller emulator that enables the user to perform instruction-level simulations. Symphony takes a different approach: instead of emulating a specific microcontroller, it models the behavior of diverse hardware components in terms of their energy consumption and the time they require to execute specific operations. The time and energy parameters for individual hardware components recreated in Symphony are derived by conducting measurements on real devices while they are performing specific operations.

Another axis of comparison would be the vast pool of tools, platforms and languages aimed at formal verification of software and distributed system level operations. The typical representatives of the modeling languages are VHDL [26], and Verilog [27] hardware description languages, and SystemC [28], the system modeling language mimicking the syntax of the previous two. The work in [29] proposes a platform for simulation of networked systems based on SystemC language. The authors demonstrate a better performance of their approach compared to that of network simulator ns2 (which is the currently obsolete predecessor of the ns3 simulator). One important remark to be made in connection to the approach presented in this article is that the above referenced modeling languages as well as the simulation facility using them must be analyzed in the context of implementation strategies of the particular operating systems. Indeed, all of early and most of the current network simulators are not suitable for formal verification purposes. At the same most of the mainstream operating systems for resource constrained computing devices are not implemented using specialized modeling languages and thus are not suitable for the formal analysis either. Symphony in this respect offers a platform for simulation-based validation of real-time properties of software running under the execution model of the particular (non-formally verifiable) operating system. In the case of Symphony the multithreaded and parallel execution of the software of different nodes are natively supported by the specialized schedulers of the ns-3 simulator [30] as well as the interfaces for interacting with the real world [31].

Interesting recent efforts on creating a platform for experimenting with networked embedded devices are reported in [32]. The direct comparison to the presented in this article approach is, however, impossible due to the differences in the functional features of the two. One of the most important things that sets Symphony apart is its use of the popular ns-3 simulator as its core platform for the orchestration and execution of simulation experiments and as a source of well-established radio propagation and physical channel models. This enables developers to experiment with holistic machine-to-machine systems that incorporate heterogeneous radio technologies, such as the communications systems of backbone networks. Secondly, Symphony uses real virtualized WSN node operating systems in its ns-3 simulations, enabling the developer to experiment with multiple different implementations of a given distributed algorithm in a single simulation. Finally, Symphony contains a set of models that accurately mimic the execution times and energy consumption of various hardware components. These features mean that Symphony simulations can accurately reproduce the behavior of real-world WSN systems.

## 3. Symphony-System Architecture

**Figure 2.**
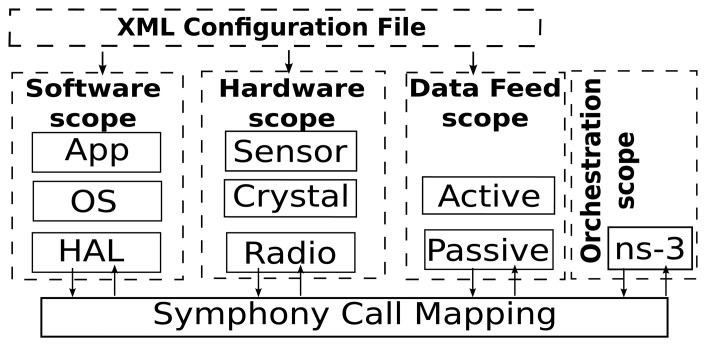
Architecture of the Symphony framework.

Figure 2 illustrates the core architecture of Symphony and its four programming scopes. The *software scope* deals with the mapping of function calls to the underlying hardware scope. The level of abstraction is configurable, and the scheduler of the underlying WSN OS is preserved. The *hardware scope* consists of a clock and a series of models for hardware components such as radio devices and sensors. These hardware models ensure that the application code is executed on a device-specific time scale. The *data feed scope* contains mechanisms for either actively or passively feeding data to the relevant software handlers or specific sensor nodes. The *orchestration scope* is implemented on top of the general purpose network simulator ns-3. It is responsible for integrating all of the other scopes with the sophisticated communication models and the event scheduling engine of ns-3 to create a holistic simulation environment. All of Symphony's operational scopes are parametrized using a single XML configuration file.

### 3.1. Models of Operating Scopes and Profiling Principles

**Figure 3.**
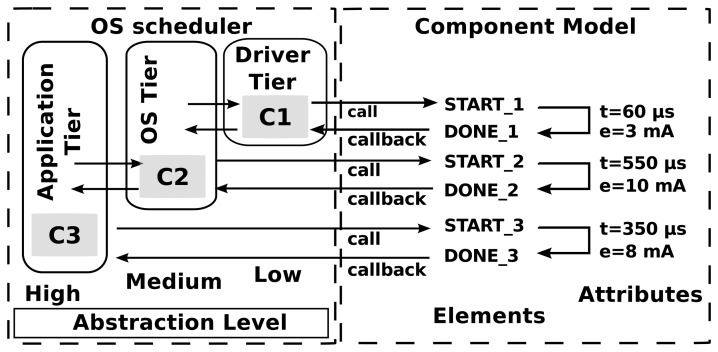
Model configuration using xml and hardware models.

In Symphony, nodes are simulated using a set of models that provide a homogeneous description of the behaviors of both hardware and software components in terms of execution times and energy consumption. Figure 3 provides a graphical illustration of this approach to modeling, while Listing 1 shows a representative part of an XML model configuration file. The figure shows three component types, *C1*, *C2*, and *C3*, which describe functionality at different levels of granularity. *C1* components correspond to the lowest level of abstraction, i.e. they represent hardware components such as a radio device and its driver. These components perform the primitive operations of sending and receiving bytes. *C2* components represent an intermediate level of abstraction, such as a function that queues packets, inspects and modifies their headers, and then transmits them onwards. Finally, *C3* components are very high-level software components; for example, a function that accept packets, encrypts and decrypts them, and performs application-specific operations before transmitting them onwards. The level of granularity in the simulation can be configured by the user. For example, it is possible to perform system-level experiments using only application-level components or, at the other extreme, to focus on low-level operations using driver-level models. Simulations of the latter sort are particularly useful for very fine-grained debugging.

The component models describe the component's time and energy properties when it is called (by a call) and when it returns control to its caller (via callbacks). The time and energy properties for a given component are defined by its attributes, as shown in Listing 1.

The component models also describe the properties of *callbacks*. These include information on the return type and the input parameters of the function. In the example shown in Listing 1, the time and energy values were determined by measuring the time required to complete a specific operation and the energy consumed when doing so for a specific device. The acquisition of such measurements is referred to as *profiling*.

Profiling is typically performed as part of a systematic measurement campaign. The best way of determining the execution time and the energy consumption of a specific component is to use external measuring equipment. We anticipate that a library of profiles for different components and platforms will be assembled over time and made available to Symphony users. The profiles discussed in this article were generated using a high accuracy 24-bit NI-4461-ADC analog to digital converter PCI card manufactured by National Instruments. The AD-card was connected to a board that was used to supply power to the node. The experiments were performed on a platform featuring a 16-bit micro controller with a maximum speed of 20 MHz, 31kB of RAM, an IEEE 802.15.4 compatible radio transceiver, several on-board sensors, and the A/D converter [33]. A range of components based on the TinyOS operating system have been profiled already to showcase the process, including a raw radio interface (representing the lowest level of abstraction), the ActiveMessage component (representing an intermediate level of abstraction), and several different security functionalities (representing the highest levels of abstraction).

While all of the showcases presented in this article use the TinyOS operating system, Symphony is a generic virtualization platform. The next subsection discusses Symphony's assembly and usage patterns, which are the same for all WSN operating systems.

Listing 1: Part of a device model in XML format describing the execution time and energy consumption of a representative component.

1<symphony>2 <scope name=hardware>3 <model name=“C1” time_unit=“micro” energy_unit=“mA”>4  <call name=“START_1” />5  <callback return=“int” params=“1” param1=“void *” time=“60” energy=“3” name=“DONE_1”/>6  …7 </model>8<scope name=software>9 <model name=“C2” time_unit=“micro” energy_unit=“mA”>10  <call name=“START_2”/>11  <callback return=“int” params=“2” param1=“uint8_t” param2=“void *” time=“550” energy=“10” name=“DONE_2”/>12  …13 </model>14<scope name=software>15 <model name=“C3” time_unit=“micro” energy_unit=“mA”>16  <call name=“START_3” time=“35” energy=“1” />17  <callback return=“int” params=“1” param1=“uint8_t” time=“350” energy=“8” name=“DONE_3”/>18  …19 </model>20</scope>21…22</symphony>

### 3.2. Details of Symphony Integration and Usage

This subsection discusses the integration of Symphony's software, hardware and data feed scopes into a cohesive whole to form a powerful and general simulation environment. The individual scopes are discussed in detail in the following sections. Essentially, the Symphony framework allows the user to seamlessly compile their software and then run it either on a real node or inside the simulation environment. In both cases, the execution model of the underlying operating system is preserved. When the software is compiled for Symphony, a binary image of a node's software is created. During the simulation's startup process, the binary file is loaded into the memory and function symbols for matching functions, which are specified in the XML configuration file, are linked to the corresponding model functions using the *dynamic linking* facilities of C++. This completes the virtualization process, enabling the node to be started inside ns-3. Within the simulated environment, the node runs according to its internal OS scheduler, preserving its original execution model. The emulated ticks of the node's internal clock are generated using Symphony's *clock model*.

In Symphony, each node model loads its own copy of the code image, therefore, the simulation framework is capable of virtualizing either *several different instances* of the same operating system or *several different operating systems* and running them within a single simulation. Practically it means that each node's code is executed independently from each other and each node can run different code. Symphony only makes calls to OS image that are specified in the XML model, for example, crystal ticks result in a 1024 calls to OS image during one second if no clock drift was initiated and timer scale set to milliseconds.

Loading isolated code image for each node model allows running simulation as in a real life scenario: software is executed by each node independently from each other and in parallel. Such solution, however, created one of the biggest technical challenges when implementing Symphony. Linux based operating systems uses *elf* − *loader* (from *glib* − *c* library) to open binary files and load them into the memory. The number of such dynamically linked files that can be opened from one application is limited to (for example, in Linux this number is currently set to 14). As a partial solution to this problem, a patched version of *elf-loader* [34] was integrated into Symphony. This makes it possible to load a substantially larger number of node images; in principle, it would be possible to open an unlimited number of static libraries. In practice, the performance of the host hardware will impose an upper boundary on this quantity.

The simulation scenarios with Symphony are constructed and executed inside the ns-3 environment. This enables experimentation with complex scenarios reusing native ns-3 modules and models, well-developed communication models, and scheduling mechanism. Technically, Symphony adds a new type of a node model and the associated infrastructure (containers, helpers, etc.), which are inherited from the base classes of ns-3. From the user perspective, however, the simulation work flow remains the same as in the standard ns-3. This is illustrated in Listing 2 on an example of a simulation with TinyOS operating system.

The OS scope is built into a static library and open from within the new node model. When opening the library (line 8 in Listing 2) an XML file of the hardware scope is consulted on which symbols for the callbacks need to be read (line 11 in Listing 2). Behind the scene when initializing the device container (line 16 in Listing 2) the model of the hardware scope is instantiated and initialized with the values from the XML file. This model actually takes care of delaying the execution and book keeping energy properties as described earlier.

Listing 2: The set-up of simulations in the ns-3 environment.

1#include <stdio .h>2… // Standard ns–3 modules are omitted.3#include “ns3/symphony–module.h”4using namespace ns3;5int main(int argc, char *argv[]) {6 …7 TosNodeContainer c; //enables TinyOS nodes8 c.Create(10, “libtossecurity .so”); // creates ten nodes with os the image to libtossecurity .so9 TosHelper wifi;10 wifi.SetStandard(ZIGBEE_PHY_STANDARD_802154); //creates wireles standard11 wifi.SetNodeModel(“tos–security.xml”);12 YansTosPhyHelper wifiPhy = YansTosPhyHelper::Default(); //creates wifi channel13 wifiPhy.Set(“RxGain”, DoubleValue(0));14 …15 TosNetDeviceContainer devices = wifi.Install(wifiPhy, c); //installs wifi devices16 TosMobilityHelper mobility;17 …18}

## 4. Software Scope

This section provides details on Symphony's software scope. Recall that Symphony does not make any short cuts when simulating WSN functionality: A real operating system is virtualized and its execution model is preserved. In essence, Symphony intercepts calls at the desired level of abstraction and delays their execution according to the profile of the corresponding component. Symphony can be used to perform simulations on three tiers: low, medium and high. Higher tiers correspond to increased granularity in the simulation and therefore more complexity. The effects of simulation granularity on Symphony's performance are discussed in Section 7; the following subsections provide further details on each tier.

### 4.1. Application Tier

The application tier is used to perform system-level simulations and represents the highest level of abstraction available in Symphony: only the highest level calls are passed through. This tier yields the fastest simulations.

User creates component profile by measuring time and energy consumed during execution of the component. Measuring an application tier component abstracts underlying complexity of the system. For example, profiling a security algorithm on high level will abstract underlying complexity such as read/writes to hardware encryption chip. Moreover, such abstraction will lose details and particularities of runtime performance and may yield results deviating from reality. Specially, this would happen if interrupt would be generated during time of component execution. For example, if a packet would arrive during the execution of particular component, in reality, the execution would be interrupted, but if application tier is used this interrupt would be visible to OS when execution of the component have ended. The measured performance of the component are described as a part of component's XML model is shown in Listing 3.

Listing 3: Part of an XML device model that describes the execution time and energy consumption associated with the software component sec_1

1<symphony>2 <scope name=software>3  <model name=“encryption” time_unit=“milli” energy_unit=“mA”>4   <call time=“60” e=“30” name =“encrypt”/>5   <callback time=“52” e=“27” name =“encrypt_done”/>6  </model>7 </scope>8…9</symphony>

### 4.2. Operating System Tier

**Figure 4.**
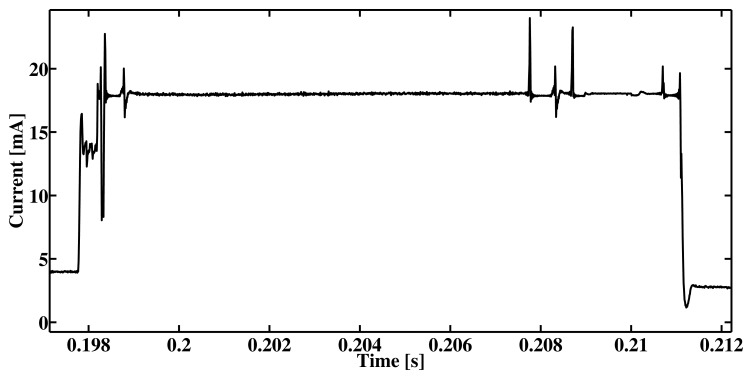
OS tier simulation: Sending 2 bytes of data with AMSend.

Operating system components are profiled in a similar way to that discussed above. This tier gives more granularity and information about system performance. Figure 4 shows the performance of the AMSend component of TinyOS when sending two bytes of data; the corresponding XML configuration file is shown in Listing 4. As seen in the figure, the measurement has fluctuation which indicates abstracted operations. For the radio transmission these operation may be turning radio on, clear channel assessment (CCA), writing to radio buffer, transmitting data etc. Using this tier in the simulation gives more detail and realism, however, the dynamic nature of the wireless sensor networks is not fully reflected. For example, the time to perform CCA in such case is a static variable which is not the case in reality.

Listing 4: Part of an XML device model describing the execution time and energy consumption for a system-level component.

1<symphony>2 <scope name=software>3  <model name=“Radio” time_unit=“micro” energy_unit=“mA”>4   <call name=“AmSend” time=“35” energy=“1” />5   <callback return=“int” params=“2” param1=“uint8_t” param2=“void*” time=“550” energy=“10” name=“AmSendDone”/>6   …7  </model>8 </scope>9 …10</symphony>

### 4.3. Driver Tier

**Figure 5.**
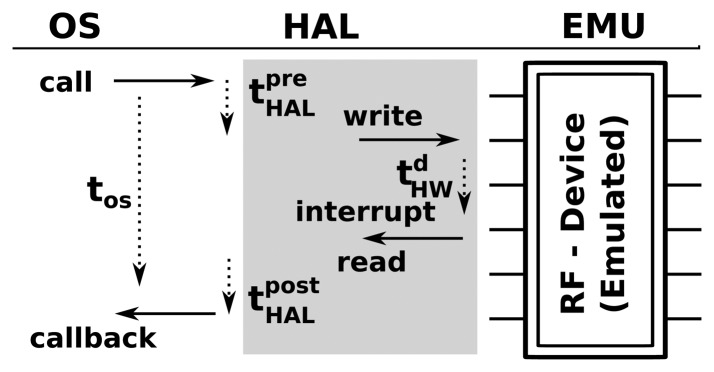
Execution time flow in hardware and emulation.

The profiling of the node on the driver tier is represented graphically by the shaded area in Figure 5. In this case, profiling is performed at the level of the hardware abstraction layer (HAL), and the execution time and energy consumption are measured for each operation of interest as discussed previously. While this tier is the most accurate one, it is also the heaviest tier to simulate due to the number of simulated calls. Using this tier may limit the number of nodes that can be simulated in real or faster time.

Listing 5: Part of an XML device model describing the execution time and energy consumption for a hardware component.

1<symphony>2 <scope name=hardware>3  <model name=“Radio” time_unit=“milli” energy_unit=“mAs”>4   <call name=“RadioSend”/>5   <callback return=“int” params=“1” param1=“uint8_t” time=“19.2” energy=“0.1” name =“RadioSendDone”/>6   …7  </model>8 </scope>9 …10</symphony>

## 5. Hardware Scope

Hardware interrupts and calls are emulated by tapping into the hardware abstraction layer (HAL) of the WSN OS. As shown in Figure 5, when an operating system makes a call to a hardware element (in this case, a call to a radio transceiver to transmit a message) in Symphony, the call is dispatched to the appropriate hardware model. Essentially, the device model is a piece of software that mimics the behavior of the real hardware component. Technically, all of the models used in Symphony are inherited from the ns3::Object class and parameterized according to the appropriate XML configuration file.

For example, a model of a temperature sensor will read temperature data (as discussed in Section 6 below) and delay the callback by the amount of time that the real device would take to perform the same operation, which is specified in the XML profile. The model of the RF230 transceiver used in the above examples can be linked to any one of the ns-3 wireless channel models.

The remainder of this section describes the implementation of a ‘crystal device’ in Symphony and its modes of operation. This component is essential for performing real-time studies of distributed embedded systems.

### 5.1. The Clock Model—Simulating Time Skew

**Figure 6.**
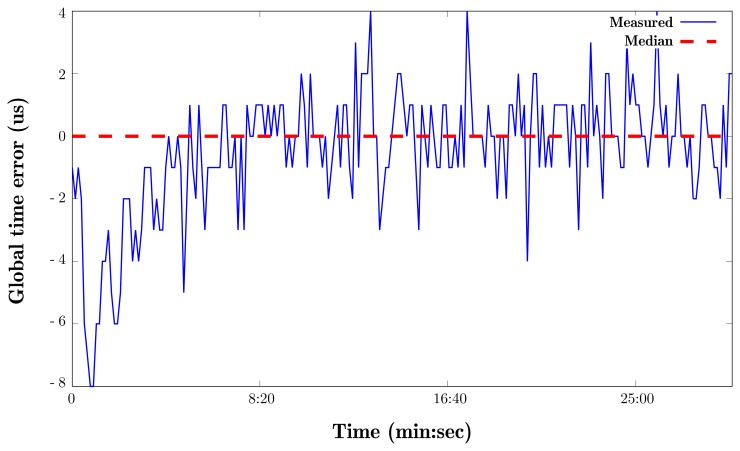
Consequences of clock drift.

**Figure 7.**
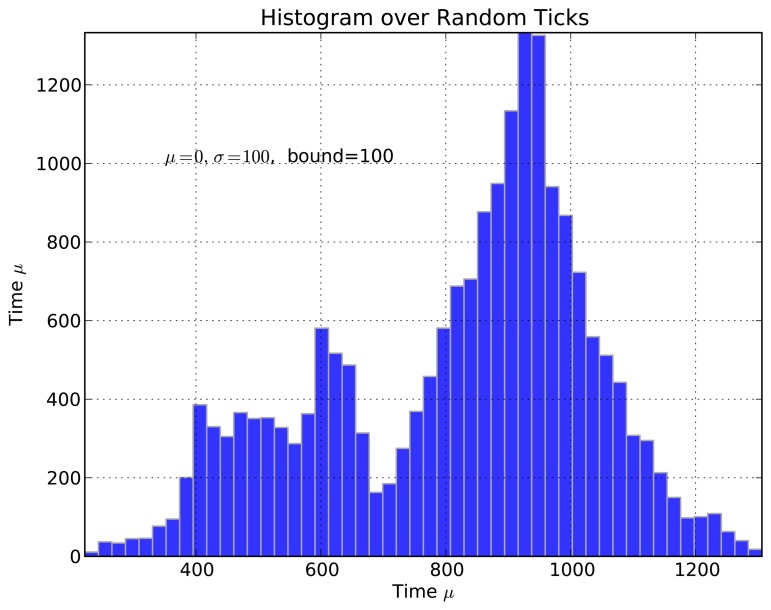
Histogram of clock ticks affected by random skew.

A typical WSN node is equipped with one or two crystals that power the device's internal clocks. These crystals generate ticks at a certain frequency and then a software counter transforms these ticks into time measurements by rounding them to a specific value. For example, TinyOS defines one second in milliseconds as 1024 ticks [35]. These clocks have a degree of drift due to differences in the oscillation frequencies of crystals from different batches. The curve in Figure 6 shows the clock drift measured on a real device. Most current network simulators lack native means of accounting for clock drift and just use simulated clocks that have no deviation (represented by the red line in Figure 7a) for all nodes. However, time skew is widely recognized to be a significant problem in WSN and has been studied extensively [36,37]. Most academics who conduct experimental work on network functionality assume that perfect time synchronization can be achieved by using *specialized algorithms or hardware* [38].

**Figure 8.**
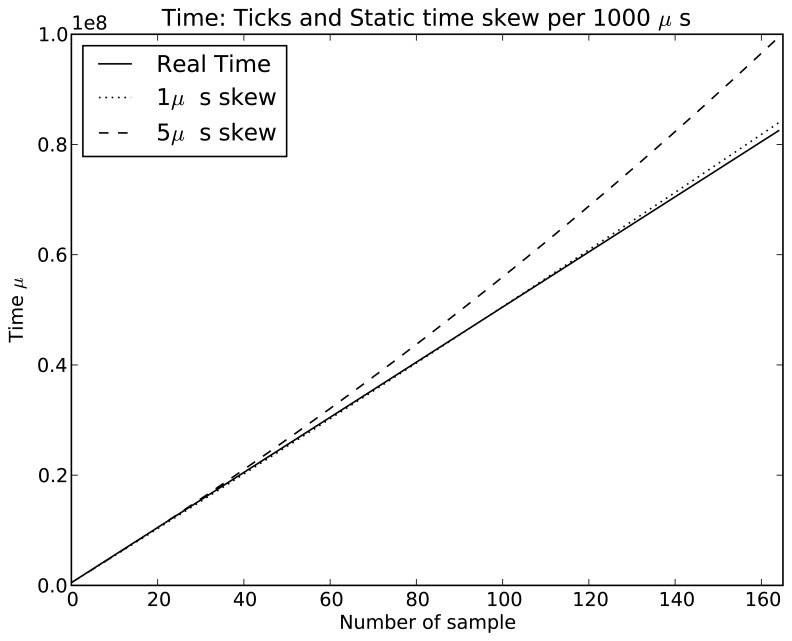
Crystal simulations: Static time skew with a period of 1000 *μ*s.

**Figure 9.**
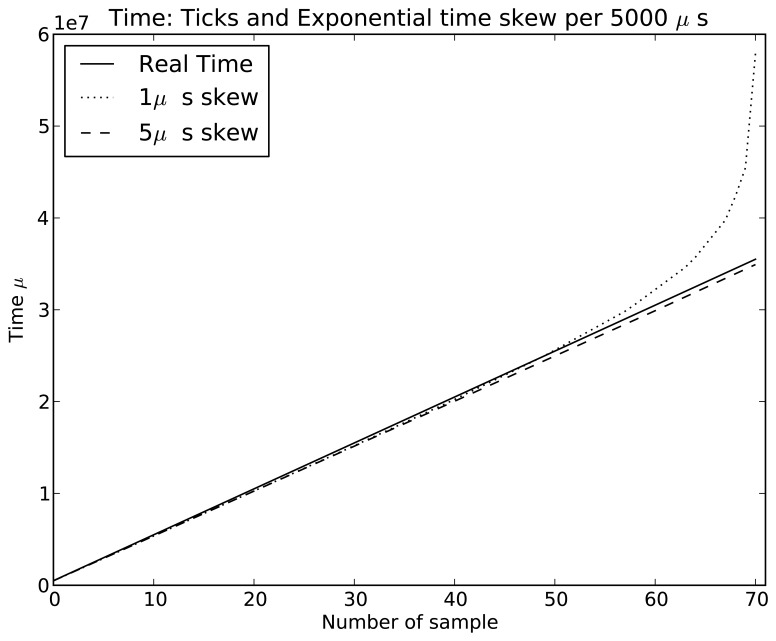
Crystal simulations: Exponential time skew.

Symphony features a native real-time clock model called *SimuClock*. When connected to an emulated node, this model generates *ticks* according to a specification provided by the user in an XML configuration file. By default, no clock drift is applied. However, the user can configure the *clocks* to drift linearly, exponentially or randomly. The random clock drift is implemented using the *Random Variable Distributions* module of ns-3. A histogram showing the number of ticks per second generated using the normal distribution is shown in Figure 7. The linear clock drift is implemented by *drifting* the clocks by a constant quantum of time as shown in Figure 8. If an exponential drift is chosen, the drift quantum doubles periodically as shown in Figure 9.

The SimuClock model can be configured in two ways: either by altering the node model description using XML as shown in Listing 6 or by modifying the ns-3 simulation file as shown in Listing 7. In both cases, the XML description follows the previously used convention: the model is defined and then the desired properties of the model are declared.

Listing 6: Part of an XML configuration file showing the parameterization of SimuClock.

1<symphony>2 <scope name=hardware>3 …4  <model name=“SimuClock”>5 <property name=“config” type=“random” drift =“1ms” randommean=“8” radomvariance=“4” driftperiod=“5ms” />6  </model>7 …8 </scope>9 …10</symphony>

Because Symphony uses ns-3 to orchestrate and execute simulations, all of its models could potentially be configured using the native configuration tools of ns-3. This is illustrated in Listing 7, which shows how one could configure the clock model using Config::SetDefault. Clock drift is disabled by default (SimuClock::NONE) and so no further configuration is required if clock drift is not desired. If drift is desired, various attributes will have to be configured as shown in the listing, depending on the nature of the drift that is required.

Listing 7: Configuration of the clock model in the ns-3 environment.

1#include <stdio .h>2… // Standard ns–3modules are omitted.3#include “ns3/symphony–odule.h”4using namespace ns3;5int main(int argc, char*argv[]) {6 …7 Config::SetDefault (“ns3::SimuClock::ClockDriftType”, EnumValue(SimuClock::RANDOM));8 Config::SetDefault (“ns3::SimuClock::ClockDrift”, TimeValue(MicroSeconds(1)));9 Config::SetDefault (“ns3::SimuClock::RandomMean”, DoubleValue(8));10 Config::SetDefault (“ns3::SimuClock::RandomVariance”, DoubleValue(4));11 Config::SetDefault (“ns3::SimuClock::ClockDriftPeriod”, TimeValue(MilliSeconds(5)));12 …13 return 0;14}

## 6. Data Feed Scope

One of the common shortcuts taken by researchers when conducting simulation-based investigations into the performance of networking functionality in wireless sensor networks is to work at a level of abstraction that does not require the consideration of real sensory data. It is often assumed that the sensory data is instantly available for transmission and that its acquisition does not in any way interfere with the sending-receiving processes. In addition, protocols are often stress tested using synthetic cross traffic.

However, in reality, the flow of sensory data through wireless sensor nodes has significant effects on the performance of all of the network's software components. In brief, before it can transmit the data, the sensor must warm up, sample the environment, pre-process the data, and packetize it. All of these operations take time. Moreover, if the data handling component is not implemented correctly, it may prevent the execution of the sending/receiving procedure and thereby violate the logic of the protocol being studied. Things become even more complicated when the external physical process sampled by the sensor is hard to adequately model mathematically (for packet generation purposes). In many cases, practitioners are most interested in problems of performance and correctness that occur under specific conditions in the physical world. None of the current network simulators allow the user to work with realistic sensory data traces. Symphony has a native tool for addressing this issue in the form of its Data Feed scope, which makes it possible to work with either pre-recorded real data traces or data that is fed into the Symphony node in real time from real hardware. These techniques introduce the possibility of performing experiments on the entire data pathway, examining the integrity of the data that is delivered to the backbone system, and experimenting with real time services based on data flows from a WSN.

**Figure 10.**
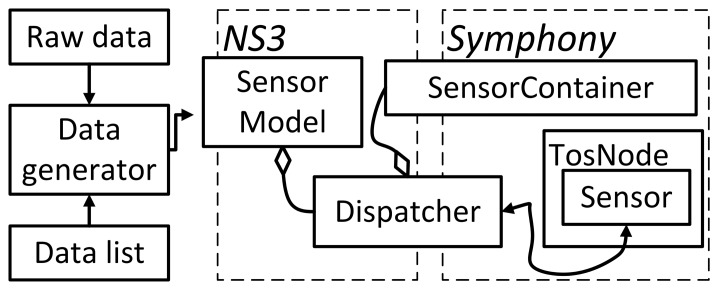
Architecture of the Data Feed scope that supports the sensor model.

The architecture of the Data Feed scope is shown in Figure 10. Symphony can handle both pre-recorded raw data and data supplied in real-time from an external generator or a numerical data list. The *Data Generator* interprets and retrieves data from specified locations. Its main purpose is to hide the details of the data retrieval process and make the sensory data available to the *Sensor Model* in a generic format. Two sensor types are supported by the model: active sensors, which issue interrupts when data becomes available, and passive sensors that release data in response to periodic polling by the OS. The *Sensor model* makes the data available to the operating system of the sensor node with delays that are specified in the appropriate configuration file. For active sensors, the model will issue an interrupt according to timestamps provided by the data generator. When the OS issues a call for a data reading to a passive sensor, the sensor model will look up the data in a list using the current time as a key.

Before the simulation begins, all nodes register their sensors with the *SensorContainer*. The *Dispatcher* block then helps in connecting the data from the *Data Generator* to the appropriate *Sensor model* of the node.

The sensor model is configured in a similar way to the other Symphony models, which are described in the preceding sections. In addition to the calls and callbacks, the sensor model has a property element that specifies the data source as shown in line 10 of Listing 4. This tells the model to read the sensory data from a user-specified file.

Listing 8: A representative sensory device model configuration file in XML format.

1<symphony>2 …3 <scope name=sensor>4 <model name=“rawsensor”>5  <callback return=“int” params=“1” param1=“uint8_t” name=“sensorStartDone”/>6  <callback return=“int” params=“1” param1=“uint8_t” name=“sensorStopDone”/>7  <callback return=“int” params=“3” param1=“uint8_t” param2=“uint16_t” param3=“void*” time=“20” units =“ms” name=“interruptData”/>8  <call name=“SplitControlStart”/>9  <call name=“SplitControlStop”/>10   <property name=“data_source” source=“/home/ubuntu/syphony/sensorydata/ temperature /” type=“file” />11  </model>12 </scope>13 …14</symphony>

The fact that the Data Feed Scope can handle read/writes from both local and remote storage (via sockets in the latter case) presents some unique challenges during implementation. This is why it was implemented as a scope in its own right rather than being treated as an aspect of the Hardware Scope.

## 7. An Experimental Showcase and Performance Metrics for Symphony

The preceding sections have outlined the key capabilities of Symphony. Given their diversity, Symphony could potentially be used to perform benchmarking studies on a very wide range of real-world communications systems in an equally wide range of scenarios. Consequently, a great deal of space would be required to present a representative selection of illustrative applications. Therefore, this section focuses on the results of a single set of experiments using different security extensions of the data packet forwarder from TOSSIM. Results obtained in Symphony simulations are compared to data from a test bed of real nodes. In addition, the runtime performance of Symphony is discussed.

### 7.1. Performance of the Showcase Scenario

We selected a case involving a computationally demanding encryption function that is known to affect the performance of various network protocols in order to demonstrate the various unique features of Symphony. The real world consequences of using this function cannot be reproduced using traditional simulation tools.

A total of six security schemes were implemented in TinyOS. A testbed consisting of 10 devices [33] was used to generate real-world results that could be compared to the simulated data. The test scenario involves a chain topology consisting of 10 nodes. The source node generates data packets of 35 bytes each. A new packet is generated when the previous one is received by the sink node. To facilitate comparisons, the total number of packets received by the sink node during the test run was counted. Each experiment was repeated ten times and an average number of received packets was calculated in each case. The same settings were used in TOSSIM, Symphony, and the testbed. The hardware scope was configured using delay and current consumption values that were determined during the execution of the security algorithms on real-world hardware while the radio transceiver was being used.

**Figure 11.**
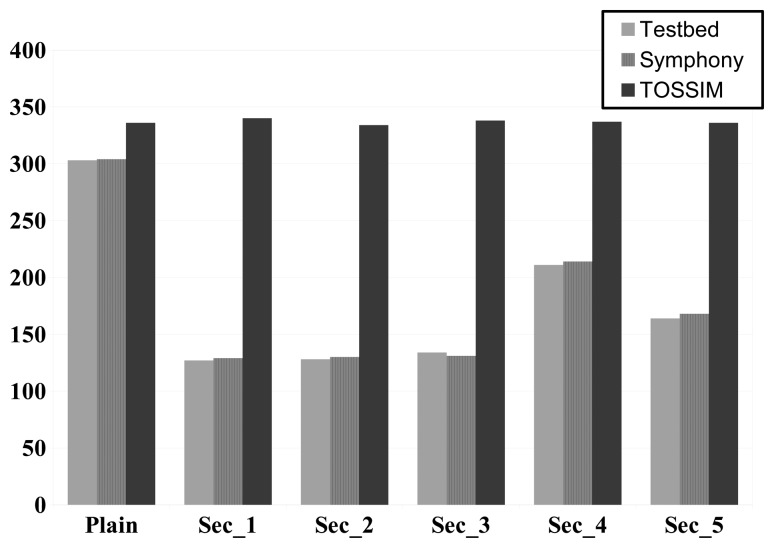
Comparison of accuracy when simulating security algorithms in Symphony, TOSSIM and experiment with real nodes. X-axes shows a comparison of different security algorithms, while on Y-axes thenNumber of received packets by sink is denoted.

The advantages of Symphony were apparent even in the simplest experiment involving communication between the nodes with no security function (the results for this case are indicated by the label “Plain” in Figure 11). The TOSSIM results over predicted the number of packets received in this scenario by 10%, whereas the Symphony results were in relatively good agreement with the data from the test bed. This is because TOSSIM does not account for the delays introduced by the hardware when transmitting data. More erroneous results were obtained when computationally intensive operations were introduced. In the experiments with the security functions, between 90% and 100% of the results obtained using TOSSIM were erroneous. This is in line with expectations because like all current WSN simulators, TOSSIM cannot account for software-induced delays. This shortcoming means that all current simulators would give completely inaccurate estimates of network protocol performance for the test case. In contrast, the Symphony results had an average accuracy of 99%. The few erroneous results generated with Symphony were attributed to the inability of the ns-3 channel model to fully describe the testbed environment.

### 7.2. Symphony's Run Time Performance

The run-time performance of Symphony depends on the mode in which the framework is operating, *i.e.*, whether it is running in real- or virtual time. In the case of virtual time operation, Symphony's performance depends on the desired granularity of the simulation, the complexity of the network topology, and the nature of the scenario. Symphony's runtime performance in real-time mode for the showcase scenario is particularly interesting because the ability to perform real-time simulations is one of the features that differentiates Symphony from other WSN simulation tools. This section therefore focuses on real-time performance results; an assessment of Symphony's performance in the virtual time operating mode will be presented elsewhere. There are two operation types that consume time during simulations and can potentially affect their accuracy: library loading and function calls in a virtualized node image. The time required to load a large number of libraries can affect the simulation bootstrapping procedure. Obviously, one needs to wait until all libraries are loaded before starting the simulation. According to the conducted measurements in scenarios with fewer than 5000 nodes this time is less than 0.5 *μ*s and, therefore, is practically negligible. Beyond this point, the loading time increases linearly with the number of nodes reaching 2.5 *μ*s in scenarios with 30,000 nodes. Symphony accounts for this behavior by using a special synchronization function to ensure that simulations start up correctly.

**Figure 12.**
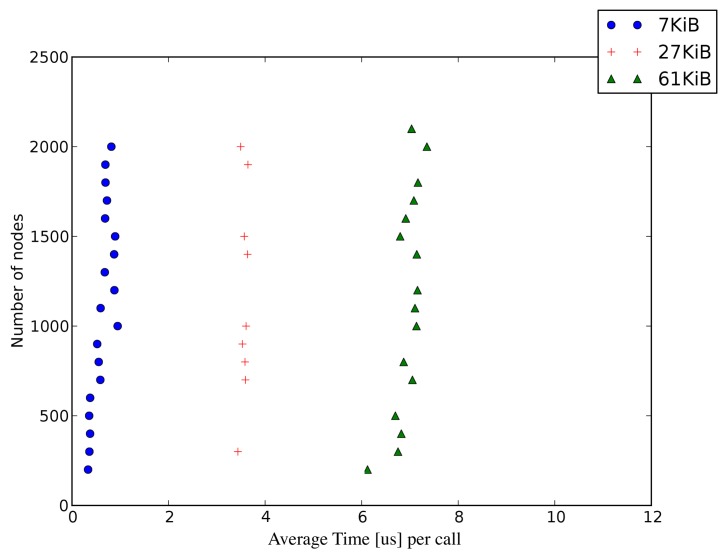
Function call time in relation to simulation granularity.

In Symphony, the granularity of the simulated model is reflected in the size of the compiled library: the lower is the abstraction level, the larger is the library. This is because the lower abstraction levels will include application, OS, and the driver-tier code, which naturally will result in a large library size. Loading large numbers of large libraries into the simulator causes frequent cache misses at the CPU level of the host machine during context switching. This inevitably increases the time required to call any function in a given library. Figure 12 shows the average time per call for different library sizes and node counts within a simulation. The call time depends only on the size of the library and not on the number of nodes.

This is a hardware-imposed limitation. While it does not affect the accuracy of experiments performed in *simulated* time, the user must account for this delay when constructing large scale *real-time* scenarios. In particular, one must ensure that the processing time for an event involving an execution chain of *n* calls can be accommodated within the real-time constraints of the application.

In practical terms, the effect of the function call time can be accounted for as follows. Suppose that we are considering an operation that takes 20 *μ*s to execute on a real hardware component, and that we wish to simulate it with a single function call to a 61 KiB library. The results shown in Figure 12 indicate that the function call time (on a host machine) for such a library is 6 *μ*s.(Symphony's performance was measured on an Intel i7 CPU with 32 GB of memory.) hardware scope, the 6 *μ*s (context-switching delay on the host machine) should be treated as part of the 20 *μ*s experimental delay that the model must reproduce. That is to say, the model will ‘automatically’ delay the simulated operation by 6 *μ*s because that is how long it takes to execute the relevant function call, so it is only necessary to add a further delay of 14 *μ*s to reproduce the experimentally observed behavior. More generally, if a component's behaviour is simulated by making *X* function calls that take *Yμ*s each then the overall call delay will be *X* × *Yμ*s, which must be accounted for when setting the execution time for the node model (which is defined in the hardware scope).

## 8. Conclusions and Future Work

This article describes Symphony, a framework for performing realistic WSN simulations. Symphony offers WSN developers seven unique capabilities: it can be used to perform experiments with the code base that would be used in a real deployment; it preserves the execution model of the underlying operating system; it makes it possible to analyze the effects of different hardware components on the performance of distributed applications and protocols; it enables experimentation with a range of time skew models; it provides a customizable level of simulation detail; and it can be used to perform experiments with real sensory data. Overall, Symphony opens new doors not only for reliable network performance evaluation and system debugging but also for experimentation with system-level WSN design ranging from backbone tests to real time service orchestration using sensory data. In the near future, Symphony will be extended with distributed computational capabilities that will be useful for extremely large-scale simulations. It will also be modified to accommodate a generic real time input-output service that will enable it to receive raw data from remote third party simulations.

## Figures and Tables

**Table 1.** A comparison of the functionality provided by selected network simulators.

**Features**	**Symphony**	**TOSSIM**	**Cooja**	**FreeRTOS ^9^**	**ns-3**
**Uses real code base**	Yes	Yes ^1^	Yes ^2^	To some extent ^3^	no
**Preserves OS execution model**	Yes	Yes ^1^	Yes	Yes ^4^	-
**Enables real-time simulation**	Yes	No	Yes	No	Yes
**Hardware emulation**	Yes, via models	No	Limited ^5^	No	Yes, via models
**Accounts for hardware-induced delays**	Yes	No	To some extent	No	No
**Incorporates energy models**	Yes	Yes ^6^	Yes	No	Yes
**Accounts for clock skew**	Yes	No	No	No	No
**Can accommodate multiple applications**	Yes	No	Yes	No	Yes
**Can be used with multiple OS**	Yes	No	Yes ^7^	No	-
**Customizable simulation detail**	Yes	No	Yes	No	-
**Realistic sensor data feed**	Yes	No	No	No	No
**Scalability**	Limited by hardware	20,000 nodes	<20,000 nodes ^8^	-	350,000 nodes
**Up to date OS**	Yes	Yes	Yes	Last updated in 2010	-

1 The real code is preserved to some extent. The node is represented as an entry in a table;

2 Provides two modes for simulation, one based on the native code and one based on simulated code;

3 The code is cross-compiled so that it can be run as a *posix* thread;

4 FreeRTOS acts as a scheduler for *pthreads* within a process;

5 Only few microcontroller and radio devices are supported;

6 PowerTOSSIM implements energy modeling. However, it is very outdated and no longer supported;

7 Cooja's emulator can load TimyOS executable compiled for platforms with MSP MCUs;

8 Fewer than 20000 simulated nodes, approximately 100 emulated nodes. The high number of simulated nodes comes at the cost of making the duration of the simulation greater than real-time;

9 Here means the simulation facility of FreeRTOS operating system.
